# Editorial: Neutrophil extracellular traps (NETs) triggered by helminths and protozoan parasites

**DOI:** 10.3389/fimmu.2025.1613886

**Published:** 2025-05-08

**Authors:** Rafael M. Mariante, Tamara Muñoz-Caro, Elvira M. Saraiva

**Affiliations:** ^1^ Laboratório de Biologia Estrutural, Instituto Oswaldo Cruz, Fiocruz, Rio de Janeiro, Brazil; ^2^ Escuela de Medicina Veterinaria, Facultad de Medicina Veterinaria y Recursos Naturales, Universidad Santo Tomás, Talca, Chile; ^3^ Laboratório de Imunidade Inata, Instituto de Microbiologia Paulo de Góes, Universidade Federal do Rio de Janeiro, Rio de Janeiro, Brazil

**Keywords:** NETs, neutrophils, protozoa, helminths, *Besnoitia besnoiti*, *Entamoeba histolytica*, *Calicophoron daubneyi*, bibliometric analysis

## 1 Introduction

Neutrophil extracellular traps (NETs) are a striking defense mechanism of innate immunity. Upon activation, neutrophils release web-like structures composed of DNA scaffolds coated with various proteins, which immobilize and kill pathogens (1, 2). However, NET formation comes at a cost, and excessive NET release can contribute to endothelial damage, as well as to autoimmune, inflammatory, and numerous other diseases in which NETs have been implicated (3). While NETs are a conserved defense strategy across species, their underlying mechanisms vary (4). Despite significant advances, questions remain regarding the role of NETs in infections caused by protozoa and helminths.

This editorial introduces a collection of articles that expand our knowledge of NETs induced by parasites. Topics include their pathological impact in amoebic liver abscesses induced by *Entamoeba histolytica*, the role of extracellular vesicles from *Besnoitia besnoiti* in NET formation and cytokine secretion, and the weak NET-inducing effect of the trematode *Calicophoron daubneyi*. A bibliometric review further highlights key research trends and future directions in the field.

## 2 NETs in infection-associated tissue damage

NETs can have both friend and foe effects on infections: they provide an important defense against invading pathogens, but excessive NET formation or impaired clearance can lead to tissue damage. In malaria, for example, a correlation has been established between disease severity and endothelial injury (5).

The study by Jorge-Rosas et al. shed light on the cytotoxic effects of NETs in *Entamoeba histolytica* infections, an underexplored aspect of amebiasis. While NETs are well known for their antimicrobial functions, this study reveals their detrimental side, showing that they contribute to tissue damage during invasive amebiasis. Using *in vitro* and ex vivo models, authors show that *E. histolytica*-induced NETs are highly cytotoxic to colonic (HCT 116) and hepatic (Hep G2) cells via serine proteases and MPO, respectively. The study also identifies NET-like structures in hamster liver abscesses, linking them to necrotic areas and implicating them in disease pathology.

These findings reinforce the idea that NETs can act as a double-edged sword in parasitic infections – trapping pathogens but also promoting tissue injury. Targeting NET components may offer new therapeutic approaches to limit tissue damage in amebiasis, a major health issue in tropical regions.

## 3 The interplay between NETs and extracellular vesicles

Extracellular vesicles (EVs) are membrane-bound structures involved in various biological processes, contributing to both physiological mechanisms – such as cell-cell communication – and pathological conditions, including cancer and autoimmune diseases (6). Recent evidence indicates that EVs are present within NETs and can modulate their release (7, 8).

In the study by Espinosa et al., EVs derived from *Besnoitia besnoiti* tachyzoites and bovine endothelial cells (BUVECs) were shown to modulate neutrophil functions. These EVs significantly induced NET formation via NADPH oxidase-independent mechanisms. While ROS production and metabolism remained unchanged, EVs were readily internalized by neutrophils. Notably, only EVs from infected BUVECs enhanced IL-1β and IL-6 secretion, pointing to distinct immunomodulatory roles.

These findings indicate that EVs from parasites and infected host cells are immunologically active, promoting NET release and cytokine production, possibly via alternative pathways such as calcium flux or MAPK. This highlights their potential role in early immune communication and suggests that EV-mediated signaling could be a target for new therapeutic strategies against bovine besnoitiosis.

## 4 Species-specific induction of NETs by microorganisms

Comparisons among protozoan groups (e.g., trypanosomatids vs. apicomplexans), between genera within the same group (e.g., *Leishmania* vs. *Trypanosoma*), or even among species within the same genus (e.g., *Leishmania* spp. or *Eimeria* spp.) reveal diverse mechanisms, constituents, intensities, and effects of NETs (9). Exploring understudied parasites offers valuable insights into the specific triggers and regulatory mechanisms behind these differential responses.

Silva et al. investigated the neutrophil response to *Calicophoron daubneyi*, a trematode increasingly affecting European ruminants. Their study assessed whether bovine neutrophils respond to *C. daubneyi* via NET formation, degranulation, ROS production, metabolic activity, and ATP consumption. The parasites induced the formation of short, weakly spread NETs, resembling the response to *Fasciola hepatica* (10), but differing from the strong NET induction observed with *Schistosoma japonicum* (11). Other responses included increased chemotactic behavior, limited degranulation, moderate activation of metabolic pathways, and increased ATP consumption.

Overall, *C. daubneyi* elicits a mild neutrophil response, suggesting it may evade robust immune detection to facilitate tissue invasion and infection. Further ex vivo studies are needed to clarify the role of neutrophils in the early pathology of paramphistomosis.

## 5 Research trends and future directions in NETs and parasites studies

In an effort to consolidate the scientific literature on NETs induced by protozoan and helminth parasites, Muñoz-Caro et al. conducted the first bibliometric analysis focused on this topic, covering publications from 2008 to 2024. The study identified key authors, institutions, countries, and journals in the field, and highlighted research trends, hotspots, and emerging themes.

After evaluating 159 original papers published across 69 journals, Germany and Brazil emerged as leading research hubs, together accounting for almost 50% of global output. Early research focused on *Leishmania*, *Toxoplasma*, *Plasmodium*, and *Strongyloides*, while more recent studies have expanded to include *Besnoitia besnoiti*, *Eimeria*, *Trichinella*, hookworms, and *Trypanosoma*. Current research is largely centered on NET formation mechanisms, parasite evasion strategies, and host-parasite interactions.

The study concluded that the field of NETs in parasitology has grown rapidly and is likely to keep expanding. It emphasized advances in understanding NET induction by parasites and their dual role in host defense and pathology. Future directions include therapeutic modulation of NETs to enhance antiparasitic responses while reducing host damage, targeting parasite evasion mechanisms such as DNase secretion, and conducting comparative studies across host species and parasite taxa.

## 6 Closing remarks

The collection of studies presented in this Research Topic underscores the complexity and dual nature of NETs in parasitic infections. By exploring both pathogenic and regulatory roles of NETs, from tissue damage in amebiasis to immune evasion by helminths, these contributions advance our understanding of how neutrophils shape the outcome of protozoan and helminth infections. Importantly, they reveal the influence of parasite species, extracellular vesicles, and host signaling pathways in modulating NET responses. We anticipate that these findings will not only inspire deeper investigations into parasite-induced NETs, but also support the development of targeted therapies that modulate NET activity to benefit the host. The integration of bibliometric insights further provides a roadmap for future research directions in this rapidly evolving field.
